# Efficacy of Subantimicrobial Dose Doxycycline for Moderate-to-Severe and Active Graves' Orbitopathy

**DOI:** 10.1155/2015/285698

**Published:** 2015-06-29

**Authors:** Miaoli Lin, Yuxiang Mao, Siming Ai, Guangming Liu, Jian Zhang, Jianhua Yan, Huasheng Yang, Aimin Li, Yusha Zou, Dan Liang

**Affiliations:** ^1^State Key Laboratory of Ophthalmology, Zhongshan Ophthalmic Center, Sun Yat-sen University, No. 54 Xianlie Road, Guangzhou 510060, China; ^2^458th Hospital of PLA, Guangzhou 510602, China

## Abstract

*Aim.* To study the efficacy and safety of subantimicrobial dose (SD) doxycycline(50 mg/d) in patients with active and moderate-to-severe Graves' orbitopathy (GO). *Methods.* Thirteen patients with active and moderate-to-severe GO received once daily oral doxycycline (50 mg/d) for 12 wk. Treatment response at 24 wk was used as the primary outcome, measured by a composite of improvement in Clinical Activity Score (CAS), diplopia, motility, soft tissue swelling, proptosis, and eyelid aperture. Secondary outcome was the change of quality of life score (QoL, including visual functioning subscale and appearance subscale). Adverse events were also recorded. *Results.* Overall improvement was noted in eight out of 13 patients (61.5%, 95% CI 31.6%–86.1%). Both CAS and soft tissue swelling significantly ameliorated in eight patients at 24 wk. Five patients (38.5%) had improvement in ocular motility of ≥8 degrees. Eyelid aperture (46.2%) also decreased remarkably. For QoL, a significant improvement in appearance subscale (*P* = 0.008) was noted during the study, whereas no difference was observed in visual functioning subscale (*P* = 0.21). Two patients reported mild stomachache at 12 wk. *Conclusions.* SD doxycycline appears to be effective and safe for the treatment of active and moderate-to-severe GO. It might serve as a new promising therapeutic strategy for GO. This trial is registered with NCT01727973.

## 1. Introduction

Graves' orbitopathy (GO) is a condition usually associated with autoimmune Graves' disease (GD), and it might be caused by immunological cross-reactivity between thyroid and retrobulbar tissue autoantigens [1]. The natural history of GO varies greatly among patients. It can either progress or spontaneously regress, and a typical process can be divided into three phases: active (inflammatory) phase, stabilization, and remission (inactive) phase [2, 3]. The active phase can last for months or even years, and this has a profound impact on patients' vision and quality of life. Therefore, the goal of treatment is to inactivate the autoimmune process. Immunosuppressive treatments are effective to arrest the active phase. So far, many immunosuppressive therapies have been reported, such as glucocorticoids (GCs), orbital radiotherapy, and cyclosporine, of which GCs represent the first-line treatment [4–6]. Oral GCs are successful in about 60% of patients with moderate-to-severe and active GO, but they often cause adverse events (81%) [7, 8]. Although intravenous (i.v.) GCs have a greater efficacy (around 80%) and adverse events are less common (39%), severe events (fatal acute liver failure and cardiovascular events) are more common [8, 9]. Other immunobiological therapies (e.g., rituximab) are currently under investigation and the efficacy and safety are not yet clearly presented [10, 11].

Doxycycline is known as a semisynthetic antibiotic. Independent of its antibiotic properties (200 mg/d), subantimicrobial dose (SD) doxycycline (40–50 mg/d; administered for a duration of 2 wk or 16 wk) displays a strong anti-inflammatory and immunomodulatory function [12, 13]. Data from clinical trials demonstrated that SD doxycycline was effective in moderating inflammation in a variety of autoimmune diseases, such as rheumatoid arthritis, multiple sclerosis, rosacea, and periodontitis [14–16]. And few minor side effects (gastrointestinal events and photosensitivity) were observed. The anti-inflammatory activities may be related to its ability of inhibiting many cytokine factors, including interleukin-1 (IL-1), tumor necrosis factor alpha (TNF-*α*), and matrix metalloproteases (MMPs), and of decreasing level of inflammatory cell infiltration and B lymphocyte proliferative responses [12, 17, 18]. Given that these cytokines and inflammatory responses are also seen among patients with active GO [1, 19, 20], SD doxycycline may serve as a treatment option. With this hypothesis, we investigated the efficacy and safety of SD doxycycline in patients with moderate-to-severe and active GO in the pilot study.

## 2. Patients and Methods

### 2.1. Patients and Study Design

From October 2012 to October 2013, consecutive patients aged 18 to 60 years, presenting with untreated active and moderate-to-severe GO, were recruited to participate in the study in Zhongshan Ophthalmic Center if they met inclusion criteria (see Supplementary Information in the Supplementary Material available online at http://dx.doi.org/10.1155/2015/285698). GO was diagnosed on the basis of typical features of the disease, such as eyelid retraction and swelling, proptosis, impaired motility, and enlarged extraocular muscles on orbital computed-tomography (CT) scans or magnetic resonance imaging (MRI) [7]. The severity and activity of GO were assessed according to the proposed criteria of the European Group on GO (EUGOGO) [21]. Patients had been euthyroid for a mean of 3 months before the date of inclusion.

All patients were treated on an outpatient basis and received 50 mg of doxycycline tablet once daily for 12 wk. We called patients every week to make sure that they took medicine exactly as ordered. Since euthyroidism was required for the study, antithyroid drugs (carbimazole, benzylthiouracil, and propylthiouracil) or levothyroxine was permitted throughout the study period, as long as the dosage was not significantly modified during the study. Also permitted were topical products (artificial tears and ocular lubricants).

The study was approved by the Institutional Review Board of Zhongshan Ophthalmic Center, Sun Yat-sen University, and conformed to the tenets of the Declaration of Helsinki. Written informed consent was obtained from all participants. It was registered on http://www.clinicaltrials.gov/ (NCT01727973).

### 2.2. Study Procedures

All patients were examined at baseline and 4, 12, and 24 wk after the start of treatment. A senior ophthalmologist with 20 yr experience of working on GO, who was masked to patients data, was asked to examine the patients throughout the study. Eye examinations were performed according to the Color Atlas evaluation (http://www.eugogo.eu/). At each visit, the subjective diplopia score was graded as no diplopia, intermittent, inconstant, and constant diplopia [22]. The eyelid aperture was measured as the distance in the midline between the eyelids, with the eyes in primary position. The lid lag was measured with a ruler as the distance between the limbus and lid margin in downgaze [23]. The soft tissue swelling was graded as no swelling, mild, moderate, or severe swelling [24]. Proptosis was measured using the same Marcus exophthalmometer and same intercanthal distance for each patient. Elevation, depression, adduction, and abduction of the individual eye were measured with a modified perimeter and recorded in degrees. BCVA was tested with a Snellen chart. The eye fundus was checked by fundoscopy. The activity of the disease was scored with the CAS. Finally, quality of life score (QoL), consisting of visual functioning subscale and appearance subscale, was evaluated with the Graves' orbitopathy-specific quality of life (GO-QoL) questionnaire (http://www.eugogo.eu/).

### 2.3. Outcome Measurements

Considering that the natural course of GO usually has an active phase lasting for 6 to 18 months, we chose the treatment response at 24 wk as the primary outcome because the response would be expected within that time frame. As for the definition of treatment response, we referred to the major and minor criteria proposed by van Geest et al. [25, 26]. The treatment response was graded as follows: improvement, deterioration, and no success (see the Supplementary Information).

The secondary outcome included the changes of GO-QoL and adverse events. At each visit, details of all possible adverse events were collected. Patients were inquired about physical discomfort and the information was noted. Changes of blood pressure, body weight, and plasma concentrations of thyroid hormones were also recorded.

In addition, the quantitative changes of proptosis, eyelid aperture, lid lag, ocular motility, and CAS after treatment were recorded and compared with baseline.

### 2.4. Statistical Analysis

Analysis was performed using the “intention to treat” method. For example, patients withdrawn from the study prematurely for any reason were included in the final analysis if the evaluation at 4 wk visit was available. Results of their last assessment were carried forward and evaluated as the last visit. Patients lost to follow-up before the visit at 4 wk were excluded from the analysis.

Baseline characteristics of the patients were expressed as means (±SD) or numbers (proportions) or median (25th to 75th percentiles), as appropriate. For parameters expressed on a continuous scale, SAS PROC MIXED (SAS Institute Inc) with time modeled as a continuous variable was used to obtain the *P*-trend over 24 wk. Due to the discrete nature of the CAS, time trends during the trial were tested with a nonparametric approach (Jonckheere-Terpstra test). Mann-Whitney *U* test was used to compare the ophthalmological variables between baseline and 24 wk. All statistical tests were two-tailed, and *P* < 0.05 was considered statistically significant. All analyses were performed with the Statistical Analysis System (SAS) software package for windows, version 9.2 (SAS Institute Inc., Cary, NC).

## 3. Results

### 3.1. Patients

68 patients (38 females and 30 males) with GO were referred for screening. Due to stringent inclusion and exclusion criteria, 20 patients were eligible and invited to participate in the study. Of these 20 patients, four declined to participate because of fear of the treatment or a wish to receive other treatments. The remaining 16 patients were administered 50 mg of doxycycline tablet daily for 12 weeks. Three patients dropped out because of immigration (one male) or lost to follow-up (two females) before the visit at 4 wk, and these three patients were not included in the final analysis. Out of the remaining 13 participants, 11 completed the study at 24 wk, while 2 patients finished the evaluation at 12 wk. Results of their last assessment were carried forward and evaluated as the last visit. The baseline characteristics of the subjects were shown in Table 1. The majority of patients were male (10/13 patients). Four patients had euthyroid Graves' disease (4/13, 30.8%), and all of them were males.

### 3.2. Efficacy

Based on predefinition criteria, 12 wk doxycycline treatment led to successful response in eight out of 13 patients (61.5%, 95% CI 31.6%–86.1%) at the end of the trial. Deterioration occurred in two males at 12 wk, with worsening of soft tissue swelling. Both patients completed the evaluation at 12 wk and were subsequently treated with oral prednisone. No success was observed in the remaining three participants.

Table 2 showed the changes in individual variables. CAS significantly decreased during the trial (*P* < 0.0001). Two patients had a decrease of three points at 24 wk, and six patients had a two-point decrease. Soft tissue swelling also improved in eight out of 13 patients (61.5%) at 24 wk. A significant improvement was found when compared with baseline (*P* = 0.044). Interestingly, the majority of patients (9/13) reported that spontaneous orbital pain was relieved at 4 wk.

Only three patients (23.1%) reported improved diplopia at 24 wk: one from inconstant to no diplopia, one from inconstant to intermittent diplopia, and one from intermittent to no diplopia. Nevertheless, five patients (38.5%) had remarkable improvement in motility of ≥8 degrees at the end of trial. By investigating the motility at different directions in detail, we found that elevation, which represented the function of inferior rectus, significantly improved (*P* = 0.0496).

Proptosis decreased in the minority of patients (2/13). No difference was observed compared with baseline. Both eyelid aperture (6/13, 46.2%) and lid lag (7/13, 53.8%) had substantial decrease at 24 wk. In particular, lid lag significantly decreased at 24 wk, compared with baseline (*P* = 0.008).

**Figure 1:**
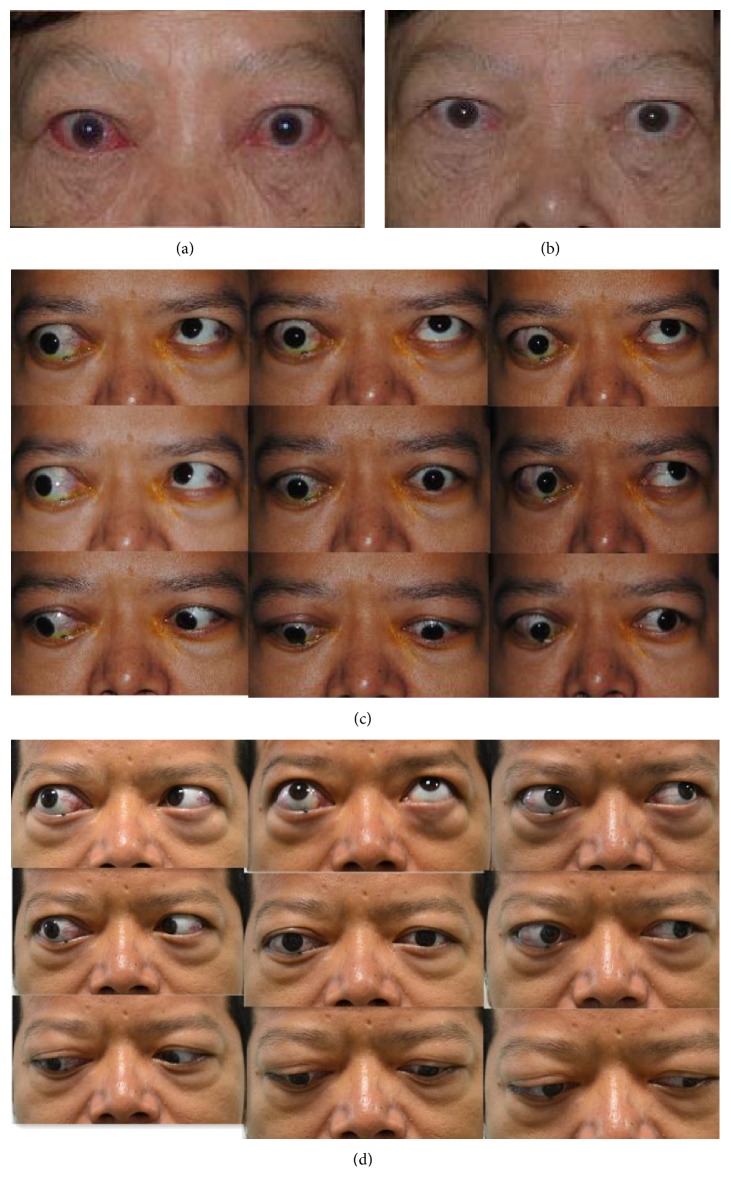
Representative example of patients status at baseline and at 24 wk after the start of treatment. (a) (baseline) and (b) (24 wk) showed that soft tissue involvements and redness of conjunctiva were remarkably improved in a female at 24 wk. (c) (baseline) and (d) (24 wk) showed the improvement of eyelid aperture, lid lag, and elevation in a male treated by doxycycline.

The mean scores on the GO-QoL questionnaire at baseline and after the intervention were also shown in Table 2. A significant improvement in appearance subscale (*P* = 0.008) was found during the study, whereas no difference was noted in visual functioning subscale (*P* = 0.21). The typical changes of appearance were demonstrated in Figure 1.

There were no differences measured throughout the study in any of the other parameters, including FT3, FT4, TSH, BCVA, corneal evaluation, and fundus examinations.

### 3.3. Adverse Events

SD doxycycline was well tolerated. No patients discontinued the study due to drug-related adverse events. Two patients reported mild stomachache at 12 wk, which disappeared when the treatment ended. No instances of vaginitis or photosensitivity were reported. Both body weight and blood pressure remained stable during the trial.

## 4. Discussion

This study is the first trial to evaluate the efficacy of SD doxycycline for patients with moderate-to-severe and active GO. An anti-inflammatory dose doxycycline (administered once daily (50 mg) for a duration of 12 wk) appeared to be effective and safe for the treatment of moderate-to-severe and active GO.

The success rate of those treated by SD doxycycline was 61.5% at the end of the trial. This was comparable to those treated by oral GCs (51–63%) or radiotherapy (60%) but lower than those treated by i.v. GCs (77–83%) [7, 8, 27]. Although i.v. GCs treatment yielded a greater efficacy, it was associated with severe adverse events, mostly consisting of fatal acute liver failure and cardiovascular events. In this study, the adverse events in those treated by doxycycline were mild and the rate (15%) was significantly lower than those treated by i.v. GCs (39%) or oral GCs (81%) [8].

It is worth noting that the proportion of patients with euthyroid Graves' disease is much higher (4/13, 30.8%) compared to data in the literature (19/221, 8.6%) [28]. Although no clear explanation can be offered for this finding, one possibility could be related to the sex distribution of patients. A study by Marcocci et al. found that patients with euthyroid Graves' disease had a F/M ratio of 0.7, whereas ophthalmopathy associated with hyperthyroidism was significantly more frequent in females, with a F/M ratio of 2.1 [28]. This indicates a higher prevalence of euthyroid Graves' disease in males. We therefore consider that the high proportion of patients with euthyroid Graves' disease in our collective may be explained by the high proportion of male patients recruited. It is unusual that the majority of our patients are males (76.9%). This is possibly due to the small number of eligible subjects recruited, since more female patients had been referred for screening. On the other hand, it confirms previous work that male subjects show severe forms of GO [29, 30]. Reportedly, female subjects respond better to anti-inflammatory therapy [29, 30]. Therefore, it stands as a chance that this study may be underpowered to detect the efficacy of doxycycline in treating GO.

Soft tissue swelling and, particularly, CAS ameliorated significantly at the end of the trial. 61.5% of patients showed an improvement of soft tissue swelling and a decrease of CAS by at least two points at 24 wk. The beneficial effect of SD doxycycline on soft tissue swelling is similar to that on those treated by GCs [7]. Moreover, nine out of 13 patients reported that the spontaneous orbital pain (one point in the CAS) diminished at the visit of 4 wk. These effects may be explained by the rapid anti-inflammatory potential of SD doxycycline. The rapid effect has also been found for rosacea, in which SD doxycycline exerted a significant reduction of inflammatory lesions within the first 3 weeks of therapy [13].

We also assessed several quantitative ocular parameters, including proptosis, eyelid aperture, and lid lag. Although proptosis showed minor change, both lid aperture and lid lag had substantial improvement. In particular, lid lag decreased remarkably in 53.8% of patients by at least 2 mm at the end of the trial. Muller's muscle hyperactivity was previously considered to account for lid abnormality, on the basis of excessive sympathetic activity of thyroid hormones [31]. Recently, Shih and his coworkers found a positive correlation between macrophage count in the Muller's muscle and severity of upper lid retraction [32]. And they concluded that the degree of inflammatory cells infiltration of Muller's muscle is associated with clinical severity of upper eyelid retraction in GO. In the current study, all patients were euthyroid, and improvement of CAS and eyelid swelling was also observed in patients with a decrease of lid lag. We suggest that the improvement of eyelid aperture and lid lag may be associated with the amelioration of orbital inflammation by doxycycline.

Additionally, we found that doxycycline demonstrated an effect in improving eye motility (5/13, 38.5%) by ≥8 degrees, but insufficiently to influence the subjective diplopia score (3/13. 23.1%). The efficacy observed in our study (38.5%) is similar to those treated by oral GCs (42%) but weaker than those treated by orbital radiotherapy (60%) [7]. The rationale for radiotherapy in significantly improving motility resides in both its nonspecific anti-inflammatory effects and inhibiting orbital fibroblasts producing glycosaminoglycans, both of which are the main actors in extraocular muscle dysfunction [33]. Considering that SD doxycycline only possesses anti-inflammatory activity, it is reasonable that the effect of doxycycline in improving motility is lower than radiotherapy.

In this study, SD doxycycline also exerted a favorable effect in improving quality of life, as assessed by the GO-QoL questionnaire. The improvement was evident in the appearance subscale but less obvious in the visual functioning subscale. This may be attributed to the amelioration of soft tissue swelling and decreased lid fissure, which reflect the changes of appearance. In contrast, visual acuity remained stable during the trial, and improved diplopia was only reported in 23.1% of patients. These two factors dominantly contribute to the visual function. Hence, it is reasonable that the visual functioning subscale was less affected.

Of note, adverse events associated with SD doxycycline are rare. Only two patients reported mild stomachache at 12 wk, not requiring additional medication. No patient complained about photosensitivity or vaginitis. These findings are in line with previous studies for patients with rosacea and periodontitis [34, 35]. By extensive investigations of the oral, gastrointestinal, genitourinary, and skin microflora of patients receiving SD doxycycline for 6 to 18 months, no reduction in the microflora was observed, and no development of resistant organisms was found [36, 37]. Unlike GCs, which frequently lead to weight gain and hypertension, SD doxycycline did not impact weight and blood pressure. Taken together, the minor side effect of SD doxycycline highlights its good tolerability in long-term usage. According to previous studies, the most common dose of subantimicrobial doxycycline used as anti-inflammatory agent is 40 mg (20 mg per tablet) [38]. Since the formulation of 20 mg for doxycycline is unavailable in China, we adopted the common formulation of 50 mg, administered once daily. The long half-life of doxycycline permits once-daily dosing. Still, it turned out that the side effects were minor.

We must acknowledge that this study bears the limitation of being an uncontrolled trial. To minimize the bias, a senior ophthalmologist, who was masked to patients data, performed the examination throughout the study. Considering that the clinical assessments and outcome measurements were objective and were largely consistent with those introduced by prior studies [5, 25, 27], we think the findings are representative. A multicenter, double-blind, randomized, and controlled trial (doxycycline versus GCs) is urgently needed to confirm its effect. Such kind of study is in progress in China, led by our group.

## 5. Conclusion

Subantimicrobial dose doxycycline appears to be effective and safe in treating active Graves' orbitopathy. This finding may suggest a new promising therapeutic agent for active GO.

## Supplementary Material

The supplementary information presented the inclusion criteria and exclusion criteria for patient selection and the primary outcome measurements.

## Figures and Tables

**Table 1:** Baseline characteristics of patients.

Characteristic	
Demographic and clinical characteristics	
Age (yr)	43.38 ± 11.82
Gender (female)	3 (23.1%)
Weight (kg)	68.08 ± 12.87
Smoking history	
Current smoker	6 (46.2%)
Ex-smoker	1 (7.6%)
Never-smoker	6 (46.2%)
History of thyroid disease	
Graves' hyperthyroidism	9 (69.2%)
Euthyroid Graves' disease	4 (30.8%)
Others	0 (0%)
Previous antithyroid treatments	
Antithyroid drugs	5 (55.6%)
Radioiodine	1 (11.1%)
Thyroidectomy	3 (33.3%)
Duration of Graves' disease (months)	15 (6–24)
Duration of Graves' orbitopathy (months)	6 (3–12)
Duration of euthyroidism (months)	3 (1.5–3.5)
Biochemical characteristics	
FT3 (pmol/L)	5.02 (4.08–6.17)
FT4 (pmol/L)	14.32 (12.56–15.80)
TSH (mU/L)	0.54 (0.04–1.01)
Eye symptoms and signs	
Proptosis (mm)	21.19 ± 1.95
Eyelid aperture (mm)	12.31 ± 2.32
Soft tissue involvements	
Absent	0 (0%)
Minimal	4 (30.8%)
Moderate	8 (61.5%)
Severe	1 (7.7%)
Diplopia (Bahn and Gorman's score)	
Absent	3 (23.1%)
Intermittent	2 (15.4%)
Inconstant	3 (23.1%)
Constant	5 (38.4%)
Clinical Activity Score	4 (3–5)
Ocular motility	
Elevation (degrees)	22.53 ± 13.07
Depression (degrees)	45.85 ± 15.97
Adduction (degrees)	41.62 ± 7.79
Abduction (degrees)	37.31 ± 11.40

FT3: free triiodothyronine; FT4: free thyroxine; TSH: thyrotropin.

**Table 2:** Eye signs, Clinical Activity Score, and Graves' orbitopathy-specific quality of life (GO-QoL) score at baseline and at 12 and 24 wk evaluation.

Variable	Baseline	Change at 12 wk	Change at 24 wk	*P* value, trend over time/baseline versus 24 wk
Proptosis (mm)	21.19 ± 1.95	0.04 (−0.6; 0.67)	−0.59 (−1.34; 0.16)	0.26^a^
Improved (*n*, %)		2 (18.2%)	2 (18.2%)	
Unchanged		11 (81.8%)	11 (81.8%)	
Deteriorated		0 (0%)	0 (0%)	
Eyelid aperture (mm)	12.31 ± 2.32	−0.15 (−1.07; 0.76)	−1 (−2.24; 0.24)	0.13^a^
Improved (*n*, %)		2 (15.4%)	6 (46.2%)	
Unchanged		9 (69.2%)	5 (38.5%)	
Deteriorated		2 (15.4%)	2 (15.4%)	
Lid lag (mm)	1.62 ± 1.94	−0.54 (−1.54; 0.47)	−2.55 (−4.06; −1.03)	0.008^a^
Improved (*n*, %)		5 (38.5%)	7 (53.8%)	
Unchanged		6 (46.2%)	5 (38.5%)	
Deteriorated		2 (15.4%)	1 (7.7%)	
Clinical Activity Score	4 (3–5)	−1.07 ± 0.95	−1.82 ± 0.87	<0.0001^b^
Improved		4 (30.8%)	8 (61.5%)	
Unchanged		9 (69.2%)	5 (38.5%)	
Deteriorated		0 (0%)	0 (0%)	
Soft tissue signs (*n*, %)				0.044^c^
Absent	0 (0%)		7 (53.8%)	
Minimal	4 (30.8%)		2 (15.4%)	
Moderate	8 (61.5%)		2 (15.4%)	
Severe	1 (7.7%)		2 (15.4%)	
Diplopia (*n*, %)				0.614^c^
Absent	3 (23.1%)		5 (38.5%)	
Intermittent	2 (15.4%)		2 (15.4%)	
Inconstant	3 (23.1%)		1 (7.7%)	
Constant	5 (38.4%)		5 (38.5%)	
Ocular motility				
Elevation (degrees)	22.54 ± 13.07	0.69 (−1.06; 2.44)	4 (0.48; 7.52)	0.0496^a^
Depression (degrees)	45.85 ± 15.97	−0.38 (−2.95; 2.19)	−1.91 (−5.97; 2.15)	0.35^a^
Adduction (degrees)	41.62 ± 7.79	−1.64 (−4.10; 0.83)	−0.09 (−3.25; 3.07)	0.78^a^
Abduction (degrees)	37.31 ± 11.40	−1.00 (−4.17; 2.17)	−1.91 (−3.87; 0.05)	0.14^a^
GO-QoL score				
Visual functioning score	61.99 ± 25.9	3.03 (−8.01; 14.07)	9.65 (−0.28; 19.57)	0.21^a^
Appearance score	46.17 ± 20.97	18.28 (7.77; 28.79)	21.59 (5.83; 37.35)	0.008^a^

^a^These parameters were expressed on a continuous scale, and repeated-measures regression model was used to evaluate the 24 wk trend over time.

^b^This parameter was presented as median (25th to 75th percentiles), and a nonparametric approach (Jonckheere-Terpstra test) was adopted to assess the 24 wk trend over time.

^c^These parameters were presented as numbers, and Mann-Whitney *U* test was employed to compare the difference between baseline and 24 wk.
