# Supplementary material for: Efficacy of Subantimicrobial Dose Doxycycline for Moderate-to-Severe and Active Graves' Orbitopathy
Source: Int J Endocrinol. 2015 Jun 29;2015:285698. doi: 10.1155/2015/285698 (PMC4499606; doi:10.1155/2015/285698)
Supplement: Supplementary file 1 — The supplementary information presented the inclusion criteria and exclusion criteria for patient selection and the primary outcome measurements. [file 285698.f1.pdf]

Supplementary information. Patient selection and primary outcome measurements

Patient selection

Inclusion criteria

a: Moderate-to-severe Graves' orbitopathy (GO), defined by having at least one of the following signs: moderate or severe soft tissue swelling; inconstant or constant diplopia in primary position; proptosis of 18 mm or more (the normal range of proptosis for Chinese is 12~15 mm, according to EUGOGO statement, moderate-to-severe was considered when exophthalmos ≥3 mm above normal range for race);

b: Active GO: Patients presenting with a CAS of three points or more;

c: Euthyroidism as indicated by normal range of free thyroxine (FT4, 9-23 pmol/l) and free tri-iodothyronine (FT3, 3.5-6.8 pmol/l) and thyrotropin concentration within or below the reference range (TSH, 0.35-5.0 mU/L). Euthyroidism can be rendered by antithyroid drugs or following thyroidectomy, or 6 months following 131-Iodine administration;

d: Patients not receiving treatments (radiotherapy, glucocorticoids, immunosuppressive agents or surgery) for their orbitopathy except for local measures.

e: Age 18-60 years.

Exclusion criteria

a: CAS < 3/7;

b: Mild GO;

c: Sight-threatening GO (including corneal breakdown and/or dysthyroid optic neuropathy). Dysthyroid optic neuropathy (DON) was considered if there was disc swelling/pallor, visual field defect, and/or best corrected visual acuity (BCVA) less than 20/30 in absence of other reasons for sign loss;

d: Patients with diabetes mellitus or other severe concomitant illness;

e: Women with pregnancy or breast-feeding their children;

f: Contraindications to the use of doxycycline.

Primary outcome measurements

Overall treatment response was graded as: improvement, deterioration, and no success.

a: Improvement, when at least one major criteria or two minor criteria were achieved, in absence of deterioration of any criterion in that observed eye. Three major criteria were: improvement in diplopia grade (disappearance or change in grade); improvement of ≥8 degrees in any direction of eye movements; reduction of three points or more in CAS. Four minor criteria were: reduction of 2 mm or more in eyelid aperture; reduction of 2 mm or

more in proptosis; improvement in grade of soft tissue swelling; decrease in CAS by at least two points.

b: Deterioration, defined as occurrence of DON, and/or worsening of soft tissue swelling, and/or worsening of diplopia, and/or an increase of ≥2 mm in lid aperture, and/or an increase of ≥2 mm in proptosis, and/or a decrease of ≥8 degrees in duction.

c: No success was defined if there was no change or the changes did not reach the improvement criteria.
